# Early life patterns of criminal legal system involvement: Inequalities by race/ethnicity, gender, and parental education

**DOI:** 10.4054/demres.2022.46.5

**Published:** 2022-01-14

**Authors:** Courtney E. Boen, Nick Graetz, Hannah Olson, Zohra Ansari-Thomas, Laurin Bixby, Rebecca Anna Schut, Hedwig Lee

**Affiliations:** 1 Department of Sociology, Population Studies Center, Population Aging Research Center, Leonard Davis Institute, University of Pennsylvania, Philadelphia, PA, USA.; 2 Department of Sociology, Princeton University, Princeton, NJ, USA.; 3 Department of Sociology and Graduate Group in Demography, University of Pennsylvania, Philadelphia, PA, USA.; 4 Department of Sociology, Brown School of Social Work, Center for the Study of Race, Ethnicity, and Equity, Washington University, St. Louis, MO, USA.

## Abstract

**BACKGROUND:**

Contacts with the criminal legal system have consequences for a host of outcomes. Still, early life age patterns of system involvement remain to be better understood.

**OBJECTIVE:**

We estimate cumulative risks of arrest, probation, and incarceration from childhood through early adulthood and assess disparities by race/ethnicity, gender, and parental education.

**METHODS:**

Data come from the Transition to Adulthood Supplement of the Panel Study of Income Dynamics (n = 2,736). We use Kaplan–Meier curves and Cox regression models to estimate cumulative risks of arrest, probation, and incarceration across the early life course and document disparities by race/ethnicity, gender, and parental education, as well as at their intersections.

**RESULTS:**

Criminal legal system involvement is common among recent cohorts, but Black and Latinx boys and young men face especially high risks. Among Black men whose highest-educated parent completed high school or less, an estimated six in ten had been arrested, four in ten had experienced probation, and four in ten had been incarcerated by age 26. Among Latinx men whose highest-educated parent completed high school or less, an estimated four in ten had been arrested and one in four had been incarcerated by age 26. Black women also experienced high risks, with an estimated one in four arrested by age 26.

**CONTRIBUTION:**

We document early life patterns of criminal legal system involvement among young people who came of age during the expansion of proactive policing and mass incarceration in the United States, providing important context for understanding the role of the system in generating and exacerbating life course inequalities.

## 1. Introduction

Proactive policing and mass incarceration have transformed the United States over the past 60 years (National Academies of Sciences, Engineering, and Medicine 2018). More than 2.2 million people are in prisons and jails across the United States (Walmsley 2018). Each year more than 10 million people are arrested (Federal Bureau of Investigation 2019), roughly 4.5 million are on probation or parole (Kaeble and Alper 2020), and millions more are stopped by police. Importantly, the reach of the criminal legal system in the United States is not evenly shared, with Black men facing particularly high rates of system involvement (Brame et al. 2014; Hepburn, Kohler-Hausmann, and Medina 2019; Wildeman and Wang 2017). Prominent socioeconomic gradients also exist, with individuals with less than a high school education experiencing the largest growth in incarceration in recent decades (Western and Wildeman 2009; Wildeman and Wang 2017).

Criminal legal system contacts have long-term negative consequences for educational outcomes (Hagan and Foster 2012), health risks (Boen 2020; Del Toro et al. 2019; Esposito et al. 2017; Sewell and Jefferson 2016), wealth (Turney and Schneider 2016), and employment (Lyons and Pettit 2011), among other outcomes. Age-specific rates of system involvement reveal that these contacts often begin early in the life course (Brame et al. 2014), suggesting that disparities in system involvement likely play a critical role in generating early life inequalities across a host of outcomes (Boen 2020; Esposito et al. 2017). Disparities in system involvement can therefore both reflect and reinforce structural inequalities, including structural racism (Alexander 2010; Brewer and Heitzeg 2008; Herring, Yarbrough, and Alatorre 2020; Mitchell and Caudy 2015; Rehavi and Starr 2012).

Despite compelling evidence of the role of the criminal legal system in shaping life chances and patterning inequality, early life age patterns of system involvement are not well documented, particularly for recent birth cohorts. Understanding life course patterns of system involvement is essential for elucidating the mechanisms producing intra-cohort inequality and informing policy and intervention efforts aimed at reducing population disparities. In support of these aims, this study uses nationally representative, longitudinal cohort data to document early life age patterns of criminal legal system involvement – including cumulative risks of arrest, probation, and incarceration – from childhood through young adulthood. We further examine racial/ethnic, gender, and educational disparities in risks. In doing so, we provide new evidence of the ubiquitous yet highly unequal presence of the criminal legal system in the lives of young people and, by consequence, their families and communities.

We build on research in this area in three ways. First, whereas most estimates of system involvement focus on incarceration, we estimate cumulative risks of arrest, probation, and incarceration. Young people who came of age during the height of proactive policing and the expansion of mass probation may have experienced high levels of system involvement, even if they were not incarcerated (Del Toro et al. 2019; Hepburn, Kohler-Hausmann, and Medina 2019; National Academies of Sciences, Engineering, and Medicine 2018; Phelps 2017). By including contacts along the criminal legal system continuum in our estimates, we provide a more comprehensive view of the reach of the system in the lives of young people.

Second, our use of nationally representative, longitudinal survey data allows us to estimate prospective age patterns of system involvement using more nuanced sociodemographic data than are generally available in administrative data. Although useful for producing population-based estimates, cross-sectional administrative data do not allow researchers to document prospective patterns of system involvement as young people age, and these data often lack detailed race/ethnicity and socioeconomic information (Eppler-Epstein, Gurvis, and King 2016). The use of longitudinal survey data allows us to assess inequalities in risks across and within multiple dimensions of social stratification across the early life course.

Finally, we assess inequalities in system involvement by parental education. Young people’s own educational attainment can be both a predictor and a consequence of system involvement (Hagan and Foster 2012), raising concerns about temporal ordering and post-treatment bias. We assess inequalities by parental education, shedding new light on the role of early life socioeconomic factors associated with risks of system contact.

In providing new estimates of early life risks of system involvement, this research offers critical context for understanding the role of the criminal legal system in generating and exacerbating inequalities in life chances, with consequences for a host of outcomes.

## 2. Data and methods

### 2.1 Data

We use data from the Transition to Adulthood Supplement (TAS) of the Panel Study of Income Dynamics (PSID). The PSID is the longest-running nationally representative longitudinal survey of individuals and families in the United States (Panel Study of Income Dynamics 2020). The TAS began in 2005 and followed young people from the original Childhood Development Supplement (CDS) cohort, who were 0 to 12 years old in 1997, as they transitioned to adulthood. TAS follows young people through age 28, but because of small sample sizes at older ages, we include only data on respondents through age 26 in our analyses. We use seven waves of TAS data: 2005, 2007, 2009, 2011, 2013, 2015, and 2017. Analytic samples include 65,754 person-years from 2,736 respondents: 1,172 non-Latinx Black (598 male and 574 female), 1,284 non-Latinx White (616 male and 668 female), and 280 Latinx (142 male and 138 female) individuals. Because of small sample sizes, estimates of risks for Latinx respondents with college-educated (some college and BA+) parents are excluded from the final results.

### 2.2 Measures

Outcomes include the ages at which young people were first arrested, on probation, and incarcerated. The TAS asked respondents in each survey wave whether they had ever been arrested, on probation, and/or incarcerated and the age at which the experience occurred. During their first TAS interview, respondents were asked about their histories of system involvement during childhood and adolescence. We use this information to backfill data about whether and at what age they had been previously arrested, on probation, or incarcerated. We are therefore able to generate criminal legal system contact histories for the TAS respondents from childhood through early adulthood.

We measure time in our study using respondent age (years). We further examine patterns of system involvement by race/ethnicity (1 = non-Latinx White; 2 = non-Latinx Black; 3 = Latinx), gender (0 = men; 1 = women), and parental education (1 = high school or less; 2 = some college; 3 = BA+), which reflects the educational attainment of a respondent’s highest-educated parent. Supplementary analyses separating less than high school from high school completion and using a measure of maternal education produced substantively similar results.

### 2.3 Methods

To estimate age patterns of risks of arrest, probation, and incarceration, we use Kaplan–Meier curves, which define the cumulative probability of experiencing contacts with the criminal legal system from childhood through age 26 while accounting for attrition and censoring. At each age, “failure” probabilities are calculated as the number of respondents who had been arrested, on probation, or incarcerated divided by the number of respondents at risk. Cumulative probabilities of failure at each age are then calculated by multiplying all the probabilities of failure at all preceding ages. Our final results show cumulative probabilities of failure to more clearly highlight how disparities in risks of system involvement evolve over age.

We examine inequalities in risks by race/ethnicity, gender, and parental education, as well as the intersections of these dimensions of inequality. To assess group differences in risks, we use Cox regression models and Wald tests, which are preferred to log-rank tests when using weighted survey data (StataCorp 2013). We highlight risks at ages 18 and 26, which represent particularly important life course transitions. (Age 18 marks the end of adolescence, and age 26 captures the transition to adulthood.) (Arnett 2000; Sawyer et al. 2012).

All estimates are weighted to correct for survey design effects using the CDS 1997 nationally representative baseline weight. The PSID provides an additional weight that includes a model-based adjustment for attrition since 1997, but this weight is not available for all TAS respondents. We conducted supplementary analyses using the longitudinal weight with the smaller non-censored sample, as well as supplementary unweighted analyses, and results were largely substantively similar to those presented here.

All study data and code are available at https://github.com/ngraetz/dem_res_2021.

## 3. Results

### 3.1 Age patterns by race/ethnicity and gender

**Figure 1:**
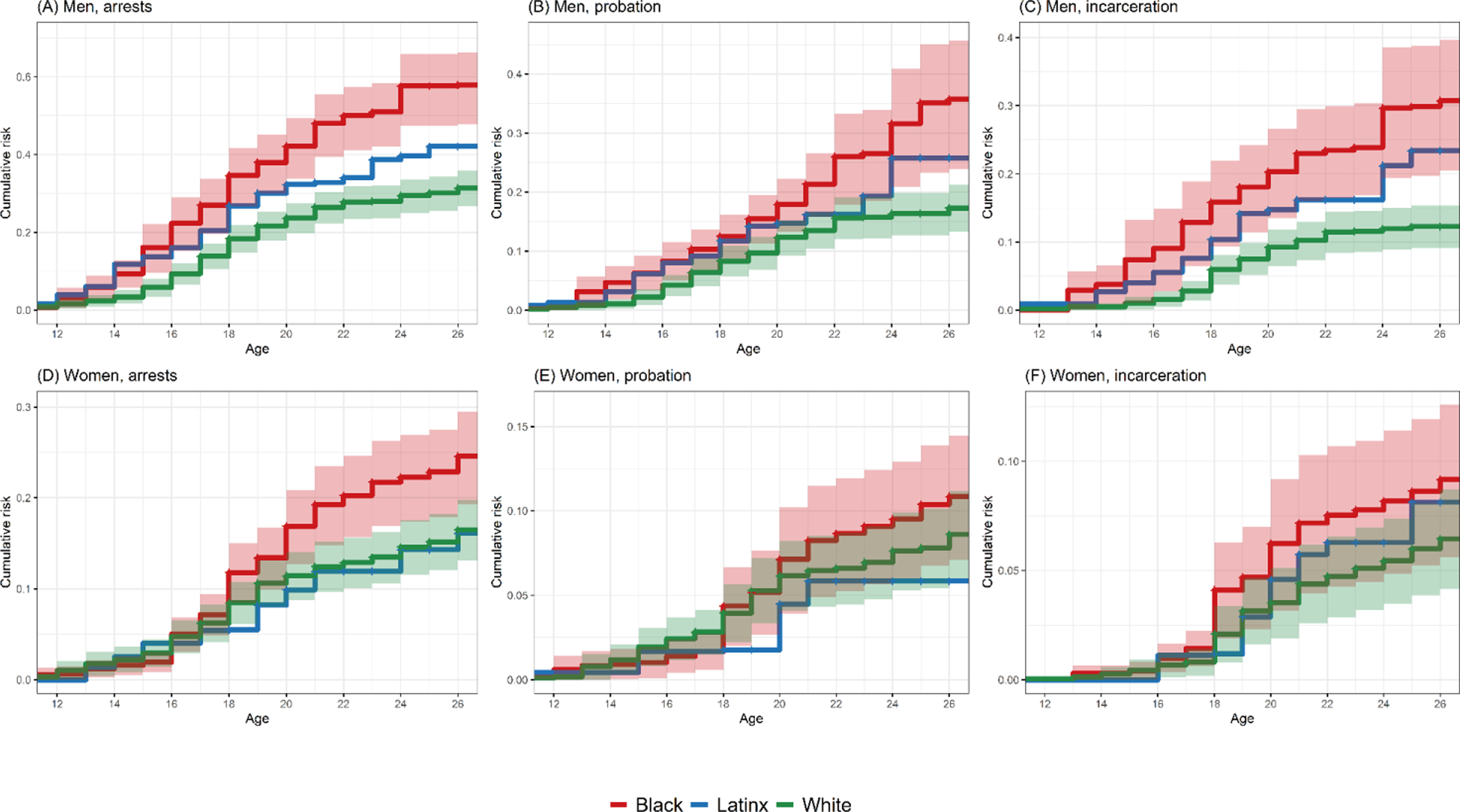
Cumulative risks of arrest, probation, and incarceration by race/ethnicity, gender, and age (with 95% CIs) *Notes*: Weighted Kaplan–Meier estimates of cumulative risks of arrest (Panels A and D), probation (Panels B and E), and incarceration (Panels C and F) by age, gender (men in Panels A–C and women in Panels D–F), and race/ethnicity; n = 65,754 person-years from 2,736 unique individuals. Shading shows 95% confidence intervals for Black and White estimates. Because of comparatively small Latinx samples, the error bounds for Latinx estimates were wide and are excluded from the figure.

Figure 1 shows Kaplan–Meier estimates of cumulative risks of first-time arrest, probation, and incarceration by age, race/ethnicity, and gender, revealing an emergence of racial/ethnic disparities in risks in adolescence that further diverges across the transition to adulthood. By age 18, young Black men are nearly twice as likely as White men to have been arrested, with an estimated 35% of Black men, 27% of Latinx men, and 18% of White men having been arrested. We estimate that by age 26, nearly 60% of young Black men have experienced arrest, a risk that is nearly double that for White men (*p* < 0.001). Risks of probation are lower than risks of arrest, but we observe similarly racially disparate risks. By age 26, an estimated 36% of Black men, 26% of Latinx men, and 17% of White men have experienced probation. Incarceration risks are also high for young Black men compared to White men (*p* < 0.001). By age 26, an estimated 31% of Black men, 23% of Latinx men, and 12% of White men have experienced incarceration. Cumulative incarceration risks of White men at age 26 (12%) are similar to cumulative risks experienced by Black men roughly eight years earlier (16% of Black men incarcerated by age 18). Results also reveal high risk of arrest for Black women compared to White women (*p* = 0.005). By age 26, an estimated 25% of Black women, 16% of Latinx women, and 16% of White women have experienced arrest. Among Black women, 11% have experienced probation and 9% have experienced incarceration by age 26.

### 3.2 Age patterns by race/ethnicity, gender, and parental education

**Figure 2:**
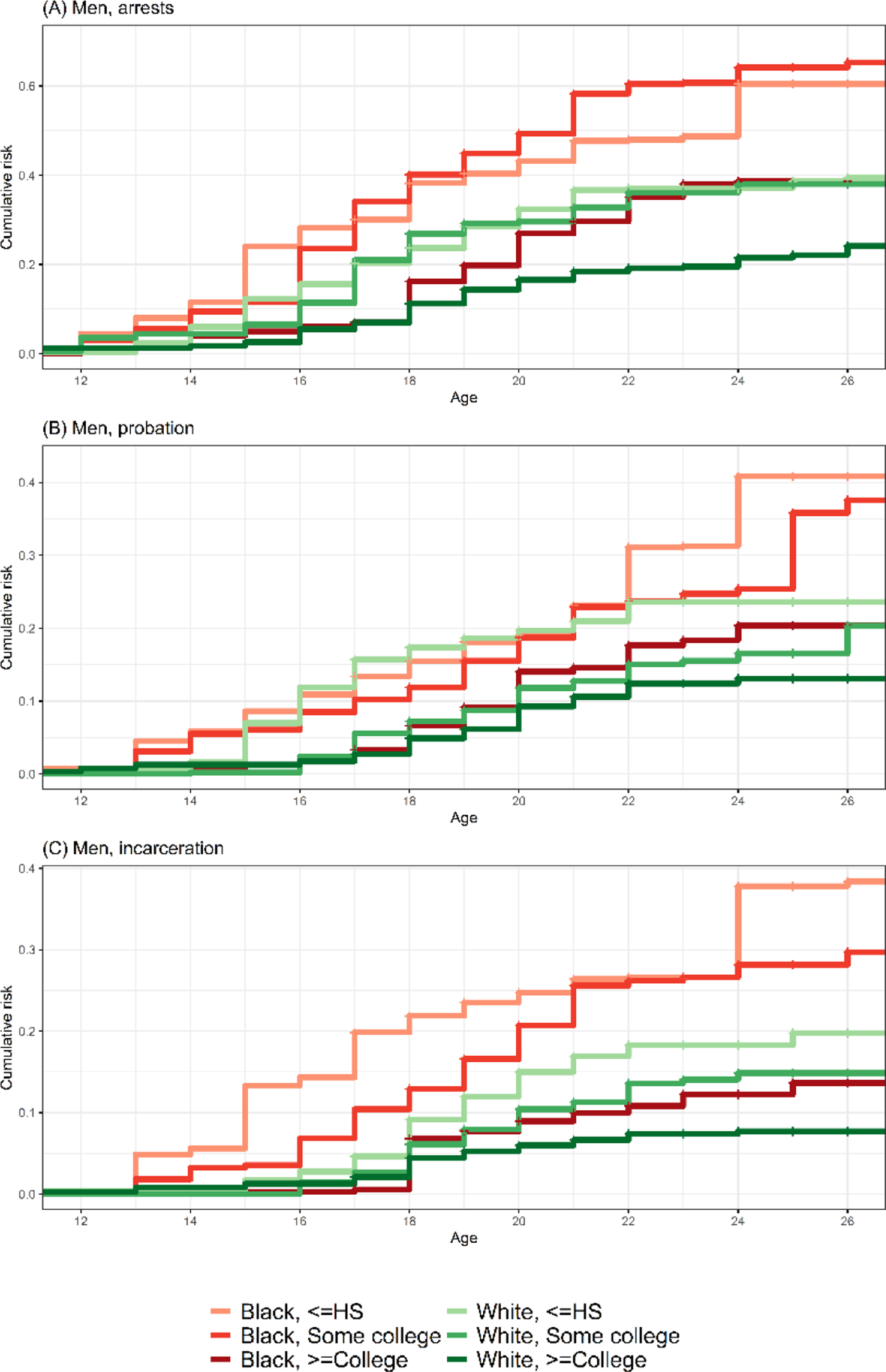
Men’s cumulative risks of arrest, probation, and incarceration by race/ethnicity, parental education, and age *Notes*: Weighted Kaplan–Meier estimates of White and Black men’s cumulative risks of arrest (Panel A), probation (Panel B), and incarceration (Panel C) by age, race/ethnicity, and parental education; n = 28,974 person-years from 1,214 unique individuals.

Table 1 displays cumulative risks of arrest, probation, and incarceration at age 26 by race/ethnicity, gender, and parental education. Figure 2 shows early life age patterns of criminal legal system involvement by race/ethnicity and parental education for Black and White young men. Taken together, Table 1 and Figure 2 reveal that while there are educational gradients in risks, there are also striking racial/ethnic disparities within educational categories, with Black boys and men whose highest-educated parent had a high school education or less facing particularly high risks.

By age 26, an estimated 60% of young Black men whose highest-educated parent had a high school education or less had been arrested, compared to 39% of White (*p* = 0.002) and 40% of Latinx (*p* = 0.048) men with similar levels of parental education. An estimated 65% of Black men whose highest-educated parent had some college education had been arrested by age 26, compared to 38% among White men whose parent had some college education (*p* = 0.016). Among men with a college-educated parent, an estimated 39% of Black men had experienced arrest by age 26, compared to 24% of White men (*p* < 0.001).

These disparities hold for incarceration. Nearly 40% of Black men whose highest-educated parent had a high school education or less had experienced incarceration by age 26, a risk that is almost double that of White men with the same level of parental education (*p* = 0.002). An estimated one in four Latinx men whose highest-educated parent completed high school or less had been incarcerated by age 26. Among men whose parent had some college education, the estimated incarceration risk for Black men at age 26 is double that experienced by White men (*p* = 0.001).

We also see disparities among women. An estimated 28% of Black women whose highest-educated parent had a high school degree or less had been arrested by age 26, which is higher than the percentage for Latinx women (*p* = 0.012). By age 26, an estimated 10% of White women, 12% of Black women, and 6% of Latinx women whose highest-educated parent had a high school education or less had been incarcerated.

## 4. Discussion

Though research highlights the critical role of the criminal legal system in shaping life chances, early life age patterns of system involvement – and disparities in risks by race/ethnicity, gender, and parental education – remain to be better understood. Using nationally representative longitudinal cohort data, this study provides evidence of the prominent but highly unequal role of the criminal legal system in the lives of young people. Recent public events and scholarship have brought attention to the role of deadly police violence in contributing to population patterns of mortality (Edwards, Lee, and Esposito 2019), but contacts with the criminal legal system need not result in death to have lasting and irreversible effects on well-being. Our results therefore provide much needed context for understanding the role of the criminal legal system in shaping inequalities in young people’s trajectories of health, development, and life chances.

Black boys and men with low levels of parental education faced the greatest risks of system involvement; among men whose highest-educated parent had a high school education or less, we estimated that roughly six in ten had been arrested, four in ten had experienced probation, and four in ten had been incarcerated by age 26. Among Latinx men whose highest-educated parent had completed high school or less, we estimated that four in ten had been arrested and roughly one-quarter had been incarcerated by age 26. While results showed evidence of an educational gradient in risks, we also showed that high levels of parental education do not translate to reductions in risks of system involvement in the same way for Black and White boys and men. Even within levels of parental education, Black men generally experienced higher risks than White men. Though overall levels of system involvement are lower for women, we still document disparities, with Black women facing particularly high risks. By age 26, an estimated one in four Black women had been arrested.

Previous research documents striking levels of incarceration in the United States – including particularly high incarceration risks among young Black men with lower levels of educational attainment (Western and Wildeman 2009; Wildeman and Wang 2017). We build on this research by also assessing risks in arrest and probation, showing that overall levels of and disparities in system involvement can be underestimated when focusing solely on incarceration. For example, among young Black and White men with low levels of parental education, we estimate that cumulative risks of arrest by age 26 are 1.5–2 times higher than cumulative risks of incarceration. These results underscore the importance of assessing contacts along the criminal legal system continuum in seeking to understand the reach of the system in the lives of young people.

Results indicate that for many young people of color from socioeconomically disadvantaged backgrounds, criminal legal contacts began in childhood or early adolescence, suggesting that these contacts can serve as critical turning points in the early life course, fundamentally shaping lifelong patterns of health and development. Given that police surveillance and carceral punishment disproportionately affect the most structurally oppressed young people, contacts with the criminal legal system can serve as a mechanism of cumulative dis/advantage, contributing to the early life emergence and divergence of inequalities across a host of outcomes.

Despite its advances, our study has limitations. First, to complement the use of administrative records, this study uses survey data on a sample of just over 2,700 young people, resulting in some relatively small subsamples (particularly among Latinx respondents). The use of longitudinal survey data allows us to prospectively follow young people across multiple life stages and assess age patterns of risk within and across individuals. The TAS also includes detailed race/ethnicity and parental education data, which is typically more limited in administrative data. There are tradeoffs between relying on relatively large samples of synthetic cohorts when using cross-sectional administrative data and relying on smaller longitudinal cohorts in social surveys. Future research should continue to use complementary data and methods to triangulate findings. Second, we use self-reported data on arrests, probation, and incarceration. Though research finds a moderate to strong degree of correlation between self-reports and official arrests (Maxfield, Weiler, and Widom 2000; Piquero, Schubert, and Brame 2014) and incarceration (Sutton et al. 2011), survey reports may be subject to reporting errors. Finally, because of data limitations, we are unable to distinguish different types of incarceration, examine police stops, test for differences in risks by birth year, assess changes in risks across other birth cohorts, or examine geographic disparities. These limitations represent important priorities for future research.

This research highlights the need for additional longitudinal data and research to illuminate the predictors and consequences of criminal legal system contacts. Importantly, our findings echo contemporary claims about the pervasive and highly unequal presence of the American criminal legal system in the lives of young people, especially socioeconomically disadvantaged youths and young people of color (Hinton, Henderson, and Reed 2018; Ghandnoosh 2015; Walmsley 2018), with consequences for a range of outcomes. Reducing inequities in young people’s life chances – and improving the well-being of young people in the United States more generally – requires critically assessing and redressing the vast reach of the criminal legal system and its surveillance and punishment of structurally oppressed communities in particular.

## Supplementary Material

Code and Images

## Figures and Tables

**Table 1:** Cumulative risks of criminal legal system involvement by age 26 by race/ethnicity, gender, and parental education (with 95% CIs)

			*By highest parental education*			
		Men			Women	
	*≤ HS*	*Some College*	*College+*	*≤ HS*	*Some College*	*College+*
** Cumulative risks** Arrest by age 26 * White*	39 (29–48)	38 (28–47)	24 (18–30)	24 (15–32)	19 (11–25)	12 (8–16)
* Black*	60 (41–73)	65 (53–75)	39 (22–52)	28 (20–36)	31 (22–40)	10 (4–16)
* Latinx*	40 (25–51)	––	––	13 (3–23)	––	––
Probation by age 26 * White*	24 (15–31)	20 (9–30)	13 (8–18)	11 (4–17)	11 (5–17)	6 (3–9)
* Black*	41 (20–56)	38 (18–52)	20 (9–30)	14 (7–21)	10 (4–15)	6 (1–11)
* Latinx*	22 (10–33)	––	––	3 (0–8)	––	––
Incarceration by age 26 * White*	20 (12–27)	15 (8–22)	8 (4–11)	10 (4–16)	9 (3–14)	4 (1–6)
* Black*	38 (18–54)	30 (19–39)	14 (4–22)	12 (6–19)	11 (4–17)	2 (0–4)
* Latinx*	25 (11–37)	––	––	6 (0–13)	––	––

*Notes*: Weighted Kaplan–Meier estimates of cumulative risks of arrest, probation, and incarceration by age 26 by gender, race/ethnicity, and parental education with 95% confidence intervals; n = 65,754 person-years from 2,736 unique individuals. Subgroup sample sizes at ages 18/26: White men: ≤ HS (n = 139/38); some college (n = 164/37); college+ (n = 289/93); Black men: ≤ HS (n = 213/55); some college (n = 199/62); college+ (n = 114/32); Latinx men: ≤HS (n = 79/24); White women: ≤HS (n = 165/58); some college (n = 174/75); college+ (n = 318/131); Black women: ≤HS (n = 265/88); some college (n = 191/82); college+ (n = 104/42); Latinx women: ≤HS (n = 76/30). Latinx respondents whose highest-educated parent had some college education or college+ are excluded from final estimates because of small sample sizes.
