# Supplementary material for: Early life patterns of criminal legal system involvement: Inequalities by race/ethnicity, gender, and parental education
Source: Demogr Res. Author manuscript; Available in PMC 2022 Mar 14. (PMC8920484; doi:10.4054/demres.2022.46.5)
Supplement: Code and Images [file NIHMS1774620-supplement-Code_and_Images.zip › dem_res_2021-master/2021_11_24/Table1_Black_College.docx]

|  | | | Male | | | Female | | |
| --- | --- | --- | --- | --- | --- | --- | --- | --- |
|  | | | High school | Some college | College | High school | Some college | College |
| arrest | 26 | White | 39 (29-48) | 38 (28-47) | 24 (18-30) | 24 (15-32) | 19 (11-25) | 12 (8-16) |
| arrest | 26 | Black | 60 | 65 | 39 | 28 | 31 | 10 |
| arrest | 26 | Hispanic | 40 (25-51) | -- | -- | 13 (3-23) | -- | -- |
| prob | 26 | White | 24 (15-31) | 20 (9-30) | 13 (8-18) | 11 (4-17) | 11 (5-17) | 6 (3-9) |
| prob | 26 | Black | 41 | 38 | 20 | 14 | 10 | 6 |
| prob | 26 | Hispanic | 22 (10-33) | -- | -- | 3 (0-8) | -- | -- |
| incar | 26 | White | 20 (12-27) | 15 (8-22) | 8 (4-11) | 10 (4-16) | 9 (3-14) | 4 (1-6) |
| incar | 26 | Black | 38 | 30 | 14 | 12 | 11 | 2 |
| incar | 26 | Hispanic | 25 (11-37) | -- | -- | 6 (0-13) | -- | -- |
| * p < 0.05; ** p < 0.01; *** p < 0.001 | | | | | | | | |
