# Supplementary material for: Early life patterns of criminal legal system involvement: Inequalities by race/ethnicity, gender, and parental education
Source: Demogr Res. Author manuscript; Available in PMC 2022 Mar 14. (PMC8920484; doi:10.4054/demres.2022.46.5)
Supplement: Code and Images [file NIHMS1774620-supplement-Code_and_Images.zip › dem_res_2021-master/2021_11_24/Table1_White_College.docx]

|  | | | Male | | | Female | | |
| --- | --- | --- | --- | --- | --- | --- | --- | --- |
|  | | | High school | Some college | College | High school | Some college | College |
| arrest | 26 | White | 39 | 38 | 24 | 24 | 19 | 12 |
| arrest | 26 | Black | 60 (41-73) | 65 (53-75) | 39 (22-52) | 28 (20-36) | 31 (22-40) | 10 (4-16) |
| arrest | 26 | Hispanic | 40 (25-51) | -- | -- | 13 (3-23) | -- | -- |
| prob | 26 | White | 24 | 20 | 13 | 11 | 11 | 6 |
| prob | 26 | Black | 41 (20-56) | 38 (18-52) | 20 (9-30) | 14 (7-21) | 10 (4-15) | 6 (1-11) |
| prob | 26 | Hispanic | 22 (10-33) | -- | -- | 3 (0-8) | -- | -- |
| incar | 26 | White | 20 | 15 | 8 | 10 | 9 | 4 |
| incar | 26 | Black | 38 (18-54) | 30 (19-39) | 14 (4-22) | 12 (6-19) | 11 (4-17) | 2 (0-4) |
| incar | 26 | Hispanic | 25 (11-37) | -- | -- | 6 (0-13) | -- | -- |
| * p < 0.05; ** p < 0.01; *** p < 0.001 | | | | | | | | |
